# A Mariner Transposon-Based Signature-Tagged Mutagenesis System for the Analysis of Oral Infection by *Listeria monocytogenes*

**DOI:** 10.1371/journal.pone.0075437

**Published:** 2013-09-12

**Authors:** Joanne Cummins, Pat G. Casey, Susan A. Joyce, Cormac G. M. Gahan

**Affiliations:** 1 Department of Microbiology, University College Cork, Cork, Ireland; 2 Alimentary Pharmabiotic Centre, University College Cork, Cork, Ireland; 3 School of Pharmacy, University College Cork, Cork, Ireland; Charité-University Medicine Berlin, Germany

## Abstract

*Listeria monocytogenes* is a Gram-positive foodborne pathogen and the causative agent of listerosis a disease that manifests predominately as meningitis in the non-pregnant individual or infection of the fetus and spontaneous abortion in pregnant women. Common-source outbreaks of foodborne listeriosis are associated with significant morbidity and mortality. However, relatively little is known concerning the mechanisms that govern infection via the oral route. In order to aid functional genetic analysis of the gastrointestinal phase of infection we designed a novel signature-tagged mutagenesis (STM) system based upon the invasive *L. monocytogenes* 4b serotype H7858 strain. To overcome the limitations of gastrointestinal infection by *L. monocytogenes* in the mouse model we created a H7858 strain that is genetically optimised for oral infection in mice. Furthermore our STM system was based upon a mariner transposon to favour numerous and random transposition events throughout the *L. monocytogenes* genome. Use of the STM bank to investigate oral infection by *L. monocytogenes* identified 21 insertion mutants that demonstrated significantly reduced potential for infection in our model. The sites of transposon insertion included lmOh7858_0671 (encoding an internalin homologous to Lmo0610), lmOh7858_0898 (encoding a putative surface-expressed LPXTG protein homologous to Lmo0842), lmOh7858_2579 (encoding the HupDGC hemin transport system) and lmOh7858_0399 (encoding a putative fructose specific phosphotransferase system). We propose that this represents an optimised STM system for functional genetic analysis of foodborne/oral infection by *L. monocytogenes*.

## Introduction

*Listeria monocytogenes* is a significant food-borne pathogen that is commonly used as a model Gram-positive pathogen for infection and immunity studies. *L. monocytogenes* causes the disease listeriosis which is acquired by ingesting contaminated food. The disease primarily affects pregnant women, the newborn and the immunocompromised. While *L. monocytogenes* infections are not frequent they have a high mortality rate (20-30%) therefore making them one of the most deadly food-borne infections [1] However, very little information is available concerning the means by which gastrointestinal colonisation and persistence occur prior to invasive disease [2]. Furthermore, it is clear that *L. monocytogenes* strains differ in their ability to cause disease with serotype 4b strains responsible for the majority of disease epidemics [2].

Therefore to investigate the early stages of intragastric *L. monocytogenes* infection we utilised the powerful molecular tool of signature-tagged mutagenesis (STM). STM is an effective technique for functional genetic analysis of microbial factors involved in the infection and colonization of a host [3]. The approach is based upon random transposon mutagenesis followed by *in vivo* selection to compare input and output mutant pools for mutants with impaired survival. Unlike sequence-based analytical approaches such as TraDIS (transposon directed insertion-site sequencing) it allows parallel physiological analysis of isolated mutant strains [4]. In STM each mutant is tagged with a unique DNA sequence to permit co-amplification of all tags from the DNA of a mixed population of mutants by a single PCR reaction [3,5]. It was initially developed to identify virulence genes in *Salmonella* *enteric* serovar typhimurium but has subsequently been used in screens in many other bacterial species [3,6,7].

The *mariner* family of transposable elements are widespread in nature and are members of the IS*630* family of Insertion sequences [8,9]. *Mos1* is the most frequently used *marnier* transposon in eukaryotes while *Himar1* has been extensively used for mutagenesis in bacteria [8]. *Himar1* was originally derived from the horn-fly *Haematobia* *irritans* and is member of the Tc1/mariner superfamily of transposable elements [9,10]. The *Himar1-*based transposon system has many advantages compared to previous transposon systems used in *L. monocytogenes*. Firstly they do not require species-specific host factors for efficient transposition and they only require the dinucelotide TA for insertion into the chromosome which is relatively common in the low-GC *L. monocytogenes* [8,9,10]. Furthermore, while previous transposon systems such as Tn*917* have a tendency to target hot-spots this is not the case with recently developed *mariner* transposon pJZ037 [11,12,13,14]. Finally transformation with mariner elements usually leads to 10-fold more mutants when compared to the Tn*917*-based vectors in *L. monocytogenes* [12].

Our STM bank was created in the *L. monocytogenes* 4b strain H7858. The *L. monocytogenes* strain H7858 is a serotype 4b frankfurter isolate from the multi-state outbreak of 1998-1999 in the USA [15]. *L. monocytogenes* serotype 4b strains are responsible for 33 to 50 percent of sporadic human cases worldwide and for all major foodborne outbreaks in Europe and North America since the 1980’s [16,17,18]. It is well established that mice offer a poor model for the analysis of oral infection by *L. monocytogenes*. Commonly used inbred strains of mice (e.g. BALB/c or C57Bl/6) require administration of exceptionally high oral doses of the pathogen in order to achieve a significant invasive infection [19]. To overcome the limitations of the mouse model we created a H7858 strain that is genetically optimised for oral infection in mice. The construction of this murinised H7858 (H7858^m^) strain was based on the previous Lmo-InlA^m^ strain created by Wollert and colleagues [20]. Our data shows that this H7858^m^ has an increased ability to infect by the oral route and will enhance the sensitivity of the STM screen, most likely through enhanced dissemination from the GI tract to mesenteric lymph nodes [21]. We have therefore created a novel STM system for use in *L. monocytogenes* which utilises a mariner-based transposon system and a murinised host strain for enhanced infection of mice via the oral route.

## Materials and Methods

### Ethics Statement

All animal procedures were approved by the University Animal Experimental Ethics Committee (AEEC) in University College Cork (approval ID 2008/32) and were carried out in a specialized facility. Work was carried out under license from the Irish Department of Health.

### Bacterial strains, growth media and reagents

Bacterial strains, plasmids and primers used in this study are listed in Table **1** and Table **S1**. All *Escherichia coli* strains were routinely grown in LB media shaking at 180 rpm at 37°C. All strains of *L. monocytogenes* were grown in brain heart infusion broth (BHI, Oxoid) or vegetable peptone broth (Oxoid) shaking at 180 rpm at 37°C. Defined media (DM) was made following the protocol of Premarante [22]. For growth curves in high salt environment 7.5% NaCl was added to BHI. Where appropriate antibiotics were added at the following concentrations: for *E. coli* 200 µg ml^-1^ carbenicillin, 15 µg ml^-1^ chloramphenicol and for *L. monocytogenes* erythromycin (ERY) 8 µg ml^-1^ and 7.5 µg ml^-1^ chloramphenicol.

**Table 1:** Strains and plasmids used in this study.

**Strains and plasmids**	**Description**	**Reference or source**
*Listeria monocytogenes*		
H7858	Wild-type strain	ATCC
H7858^m^	H7858Δ*inlA* with *inlA* locus recreated containing S192N and Y369S in this chromosome	This study
*Escherichia coli*		
XL1-Blue	hsdR17*,* supE44*,* recA1*,* endA1*, gyrA*46*, thi,* relA1*, lac*/F′[*proAB* ^+^ *, lacI* ^q^, *lacZ* M15::Tn10(*tet* ^r^)]	Stratagene
EC10B	*E. coli* DH10B derivative, with *repA* integrated into the *glgB* gene. Kan^r^	[24]
Plasmids		
NZ9000+pNZ8048b*inlA* ^m^	Internalin A containing S192N and Y369S in pNZ8048b.	[23]
pORI280	RepA^-^ gene replacement vector, constitutive *lacZ*, 5.3 kb, Em^r^	[70]
pVE6007	Temperature-sensitive helper plasmid, supplies RepA in *trans*	[71]
pORI280-inlA^m^	Internalin A containing S192N and Y369S mutation	[23]
pJZ037	*Himar1*-based transposon delivery system with pSpac(*hly*) promoter	[14]

### Creation of murinized H7858^m^ and non-polar mutants

A 2 Kb fragment was PCR amplified (primers IM466 and IM490) from the appropriate mutated pNZ8048b*inlA* plasmid, with primer design incorporating the first 16 nt upstream of the *inlA* GTG start codon [23]. The amplimers were digested with *Nco*I/*Pst*I, ligated into complementary digested pORI280 and transformed into *E. coli* strain EC10B (Table 1). The plasmids pORI280 and pVE6007 were co-transformed into H7858ΔinlA and mutagenesis preformed as described previously [24]. The reconstruction of the *inlA* locus was identified by colony PCR (primers IM317 and IM318) with the integrity of the gene confirmed by DNA sequencing.

#### Caco-2 invasion assays

Human (Caco-2) colonic epithelial cell lines (originally obtained from the American Type Culture Collection, Rockville, MD) were routinely cultured at 37°C in 5% CO_2_. Media was composed of DMEM glutamax, 10% FBS, Pen/Strep and 1% non-essential amino acids with all cell culture media purchased from Gibco. An overnight culture *of L. monocytogenes* was diluted down to OD_600_ 0.1 and grown to OD_600_ 0.8-1.0 and diluted down to cfu ml^-1^ 1 x 107. Caco-2 cells were seeded at 1 × 10^5^ cells, until confluency in 24 well plates (Falcon) and *L. monocytogenes* was infected at MOI of 10:1. On the day prior to use, antibiotics were removed from the media. On the day of use, cells were washed twice with DMEM to remove FBS. Both cell types were subjected to bacterial invasion for 1 h at 37°C in 5% CO_2_, washed once with Dulbecco’s PBS (Sigma) and then overlaid with DMEM containing 10 µg ml^-1^ gentamicin for 1 h. Monolayers were washed a further three times with PBS to remove residual antibiotic and then lysed with 1 ml of ice cold sterile water. Bacterial cells were enumerated by serial dilution in PBS and plated on BHI agar.

### Production of the STM tags

A pool of single stranded 99 bp DNA molecules containing a unique 40 bp region flanked by two invariant repeats were generated by oligonucleotide synthesis (MWG-Eurofins). The oligonucleotide tag was similar to RT1 designed by Hensel et al., except that *Xho*I was introduced at the either end of the sequence and the variable portion was flanked by *Nar*1 restriction sites [3]. Double stranded DNA tags were generated by PCR amplification using RT1 as the template and J3 and J4 as primers. The PCR was carried out in a final volume of 100 µl containing 200 pg of RT1, a 100 pmol of primers and was amplified using Go-Taq® Green master mix (Promega) under the same conditions described by Hensel et al. [3], PCR products were PCR purified (Qiagen) and digested with *Xho*I (Roche). The plasmid pJZ037 was also digested with *Xho*I and PCR purified after digestion. The PCR product was ligated into pJZ037 using T4-DNA ligase (Roche) and was introduced into *E. coli* XL1-Blue (Stratagene) by electroporation according to the manufactures instructions. Clones carrying tagged pJZ037 were screened by colony PCR by using primers pJZ037FP and pJZ037RP. A series of 60 randomly chosen tagged plasmids were checked by sequencing (MWG-Eurofins) using pJZ037FP and confirmed the hypervariability of the 40 bp central portion (data not shown).

### Generation of STM mutant banks

Electrocompetent *L. monocytogenes* organisms were prepared as previously described with the exception that vegetable peptone broth (Oxoid) was used instead of BHI to increase electroporation efficiency [25]. Approximately 1.5 µg of pJZ037 containing the STM tag was used to electroporate each 50-µl aliquot of electrocompetent cells. Bacteria were recovered in 1 ml of vegetable peptone broth-0.5 M sucrose left for 1 hour at 30°C and plated onto BHI plates containing 8 µg ml^-1^ ERY. Plates were incubated for 48 h at 30°C (the permissive temperature) and then replica plated onto BHI ERY plates and incubated overnight at 42°C (the nonpermissive temperature) to cure the plasmid.

### Infection of mice

The pools were prepared in two steps. First 48 mutants were grown individually in 120 µl of BHI-ERY at 37°C with agitation in 96-well plates. Then, a 100 µl fraction from each mutant was collected and mixed into 100 ml of BHI-ERY and grown at 37°C at 180 rpm overnight. For oral inoculation, overnight cultures were centrifuged (7000xg for 5 minutes), washed twice with PBS and resuspended at 5x10^10^ cfu ml^-1^ in PBS containing 100 mg ml^-1^ CaCO_3_. Balb/C mice were intragastrically gavaged with 100 µl inoculum. Mice were euthanized after 1 day with the mesenteric lymph nodes, spleen and livers aseptically removed. The organs were homogenized and half was used to inoculate an overnight culture containing BHI-ERY and left grow at 37°C at 180 rpm. This was then used for chromosomal DNA preparation. Chromosomal DNA was prepared using the Gene Elute Bacterial Genomic DNA kit (Sigma-Aldrich). Once attenuated mutants had been identified a second screen was carried out to verify these results but a smaller pool size was used of only 24 mutants per pool.

### Identification of attenuated mutants

Chromosomal DNA from each culture generated was extracted prior to infection of the mice for the input pool. The attenuated mutants were identified by carrying out 2 rounds of PCR. The first round used primers pJZ037 FP and pJZ037 RP which amplified at 250 bp region on the plasmid which contained the unique 40 bp region. This PCR product was then used as the template for the second round of PCR which amplified a 200 bp region. The primers used were pJZ037 FP and a unique primer specific to each STM. The primers were designed based on the sequence data from the 60 STM analysed (MWG-Eurofins), they were designed to have the same annealing temperature and the same sized PCR product.

### Identification of the transposon insertion site in the *Listeria* genome

Chromosomal DNA of 1.5 ml overnight culture was extracted using the Gene Elute Bacterial Genomic DNA kit (Sigma-Aldrich). To identify the sites of transposon insertion, we initially performed arbitrary PCR to amplify the DNA sequences flanking the transposon based on the method by Cao and colleagues [12]. DNA was amplified from either end of the transposon with a series of two rounds of PCR with Taq polymerase in the first round and KOD High Fidelity polymerase (Novagen) in the second round. In each round, a transposon-specific primer and an arbitrary primer were used. In the first round, DNA fragments from the right end of the transposon were amplified with primer pairs Marq207/JZ-001. For the second round, 1 µl of the first round of PCR was used in a 25-µl reaction. DNA fragments from the right end of the transposon were amplified with primer pairs Marq208/JZ-002 or Marq208/JZ-003, respectively. The PCR products were PCR purified (Qiagen) and transformed into TOPO plasmid pCR2.1 following the manufactures instructions (Invitrogen). The plasmid was purified and was sequenced using M13 reverse primer (MWG Eurofins). The sequence data was analyzed by both BLASTn and BLASTx at the National Centre for Biotechnology (NCBI). To verify the results from the BLAST analysis the mutants were amplified using a primer from the gene of interest and JZ-184 or JZ-185 primer corresponding to a region on the mariner insertion site.

### Bile growth experiments

For bile broth assays, overnights were grown in BHI shaking at 180 rpm at 37°C. Cells were then washed twice in PBS and inoculated into BHI containing 1% bovine bile (pH 5.5) at an approximate level of 2 x 10^5^ cfu ml^-1^. Cell growth was determined using viable cell counts by diluting cultures in PBS solution and enumeration on BHI agar. Where bile was used as the growth medium, all growth curves were carried out using manual plate counts after 8 hours of growth.

### Survival in synthetic gastric fluid

To determine the ability to survive the gastric environment, overnights were grown in BHI shaking at 180 rpm at 37°C. Cells were then washed twice in PBS and resuspended in the same volume of synthetic gastric fluid (pH 2.5) [8.3 g l^−1^ proteose peptone, 3.5 g l^−1^d-glucose, 2.05 g l^−1^ NaCl, 0.6 g l^−1^ KH_2_PO_4_, 0.11 g l^−1^ CaCl_2_, 0.37 g l^−1^ KCl, 0.05 g l^−1^ bile, 0.1 g l^−1^ lysozyme and 13.3 mg l^−1^ pepsin; adjusted to pH 2.5 with 1 N HCl [26]. Cell survival was determined using viable cell counts by diluting cultures in PBS solution and enumeration on BHI agar. Samples were taken after 2 hours of exposure.

### Statistics

Statistical analysis of data was performed using unpaired student t-tests to compare datasets with individual controls as appropriate.

## Results and Discussion

### Creation of a murinized H7858 strain with increased ability to infect mice by the oral route

**Figure 1:**
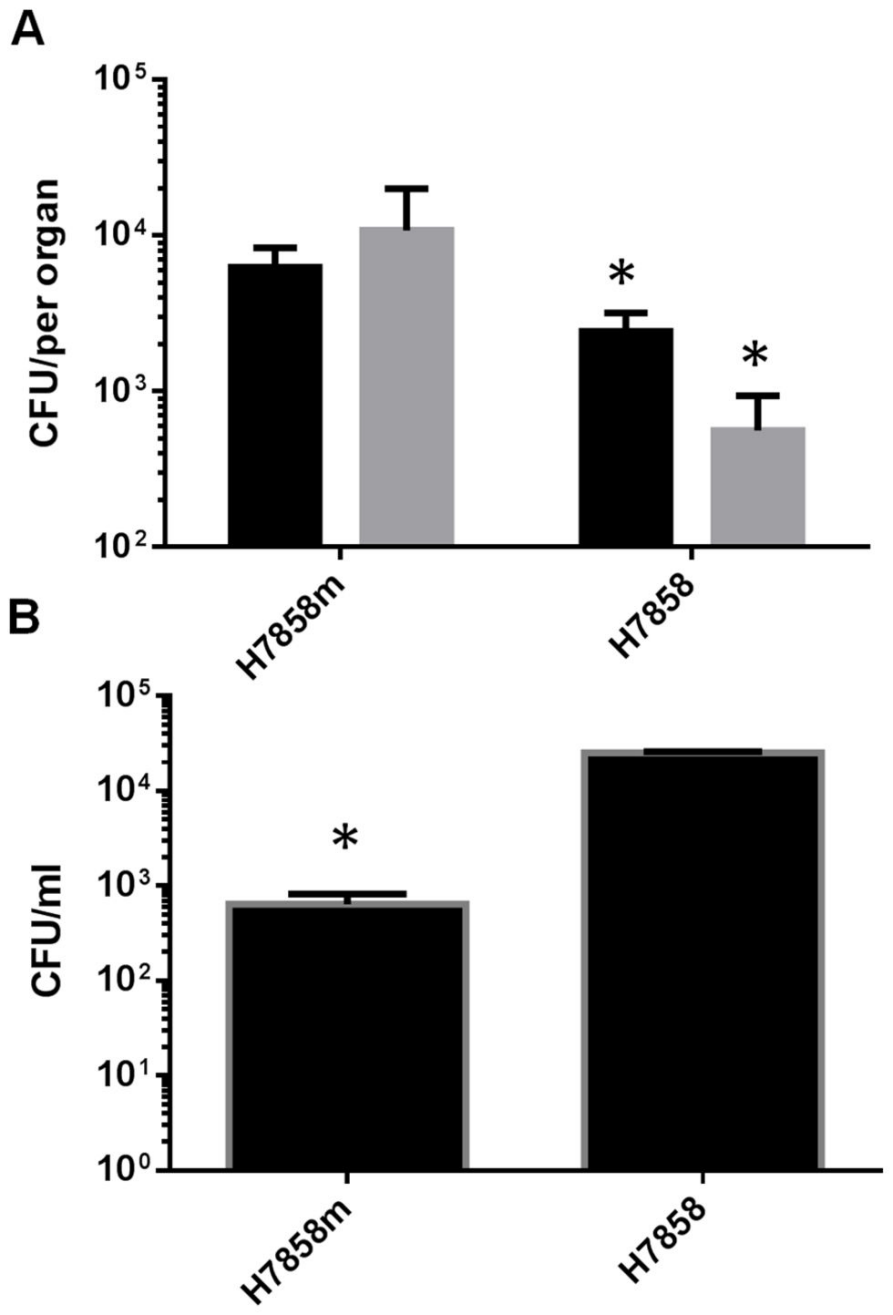
Analysis of murinized H7858 *L. monocytogenes*. (A) The murinized H7858 strain has a greater ability to infect the mouse by the oral route compared to the wild-type strain. BALB/c mice were orally infected with 1 x 10^10^ CFU with either the murinized and wild-type H7858 strain. Bacterial CFU in the liver (black bars) and spleen (grey bars) were enumerated at 3 days post-infection. N=5 mice per group and the values are the mean and standard deviation. (B) Invasion assay of Caco_2_ cell line by wild-type and murinized H7858. Under our conditions tested the murinized strain had a decreased ability to invade the Caco_2_ cell line. This was carried out in triplicate and the values are the mean and standard deviation. * indicates P<0.05 relative to control strain.

Prior to creating the STM bank we sought to improve the ability of our strain to infect mice by the oral route. We chose the 4b strain H7858 for the STM background as 4b serotypes are the most common strains associated with outbreaks and sporadic cases of listeriosis [27]. The murinized H7858 (H7858^m^) strain was created using the same alterations as previously described by Wollert and colleagues except that we utilised preferred *Listeria* codons for the mutated 192Asn and 369Ser as described by Monk et al. [20,23]. To ensure the InlA alterations had the same effect as previously reported in the EGDe background we tested its ability to infect mice by the oral route by competitive index (CI) assays. Enumeration of livers and spleens 3-days post-infection confirmed that the H7858^m^ had an increased ability to infect by the oral route compared to the wild-type strain (Figure **1A**). The H7858^m^ exhibited a 1-log increase in the number of bacteria recovered from the liver and 2-log increase in the CFU recovered from the spleen (Figure **1A**). However the H7858^m^ strain did not demonstrate enhanced invasion into Caco-2 cell line but had a decreased ability to invade when compared to the wild-type background (Figure **1B**). This is similar to findings in the recreated *L. monocytogenes* EGDe InlA^m*^ strain by Monk and colleagues [23]. The reason for this decrease is not known but it does not seem to affect the ability of the strain to infect mice by the oral route.

### Construction of STM mutant bank in H7858^m^ and *In vivo* screening

**Figure 2:**
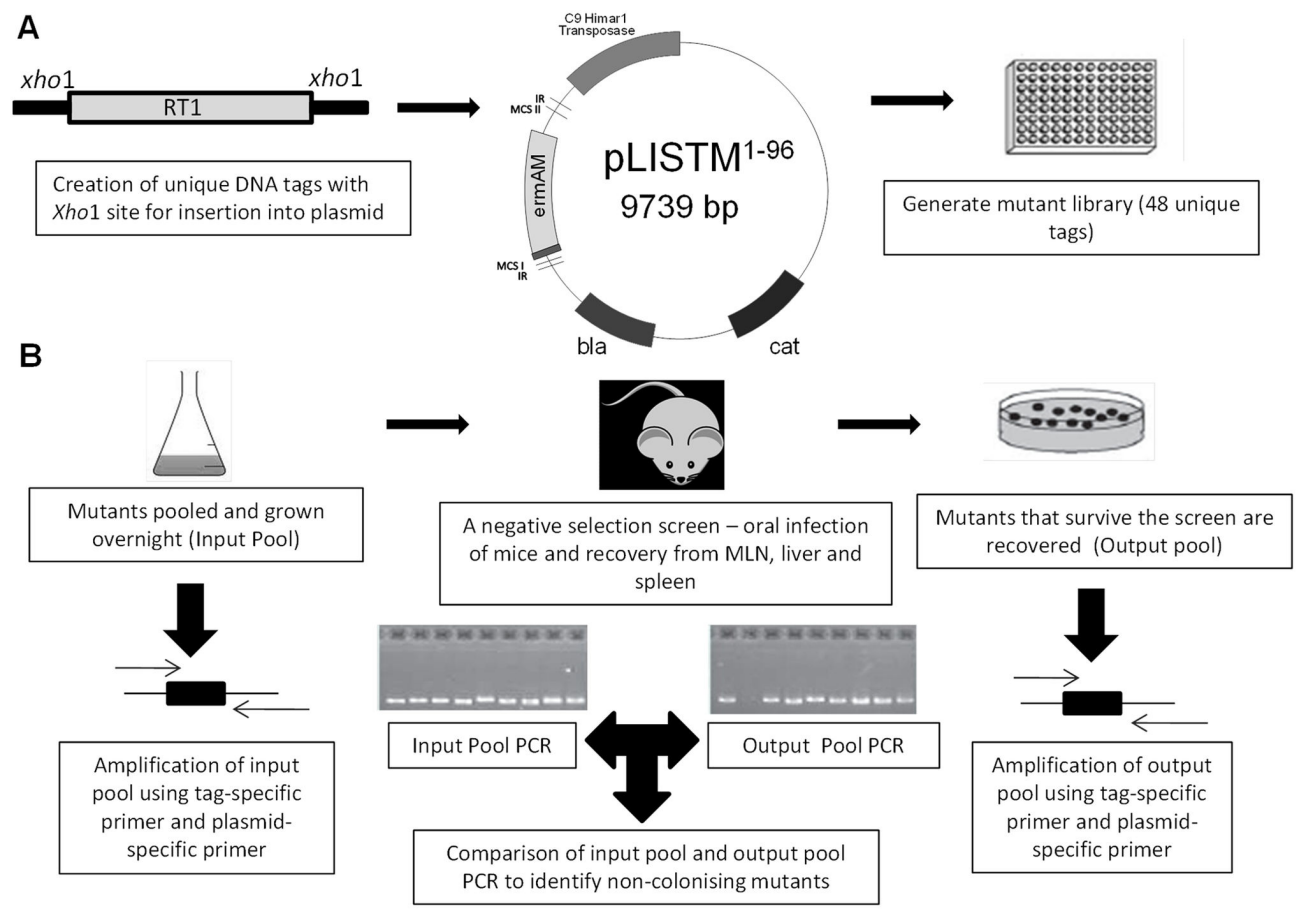
Overview of the STM system. (A) A unique STM tag was created with Xho1 restriction enzyme sites and integrated into the mariner plasmid pJZ037. In total there were 48 unique tags created in an *E. coli* background and then transformed into the *L. monocytogenes* H7858m strain. (B) The mutants were pooled and screened in BALB/c mice where the liver, spleen and mesenteric lymph nodes were removed at 1 day post-infection. The IP and OP pools were analysed by PCR to identify non-colonising mutants.

We used the *Himar-1* based transposon delivery system, pJZ037 to construct the STM system in *L. monocytogenes*. We used a mariner based transposon as it requires no factors for transposition. Rather it requires the dinucelotide TA for insertion and this minimises the potential for multiple insertions within the same region [12,14]. Double-stranded DNA tags were cloned into the *Xho*1 site of pJZ037, this site was chosen as this is the region that inserts into the host genome. The recombinant clones in *E. coli* were screened by colony PCR using primers flanking the *Xho*1 insertion site. In total 96 tags were created to ensure as much variability in the sequences as possible. They were introduced into *L. monocytogenes* by electroporation, thus generating 96 banks of *L* *. monoctyogenes* mutants (Figure **2**). A preliminary screen was performed to determine which size bank was required to ensure all STMs were equally represented. A STM bank size of 72, 48 and 24 were pooled and infected into mice as described below and from this it was determined that a bank size of 48 was sufficient to ensure all mutants were fairly represented.

**Figure 3:**
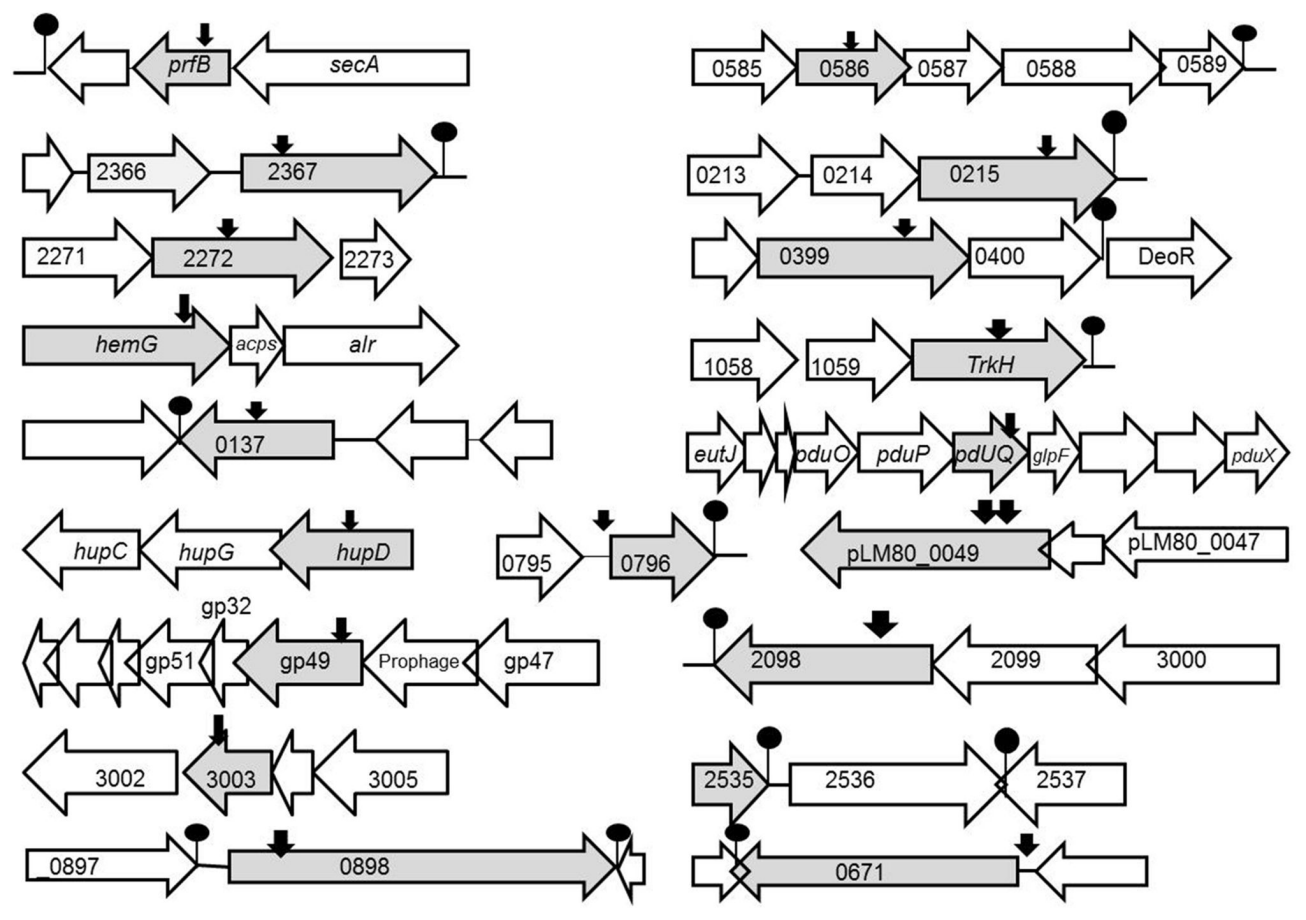
Insertion sites of transposon mutants identified in the GI STM screen. The diagram was drawn approximately to scale using *Listeria monocytogenes* H7858 genome sequence data (TIGR). Open reading frames (shaded in grey) are genes with transposon insertion. Black arrowheads represent the approximate location of transposon insertion. White open reading frames are flanking genes. Lollipops indicate predicted terminator locations. The number correspond to the lmOh7858 annotated numbers in the H7858 genome.

In this study we used STM to identify transposon insertions that decrease early *in vivo* survival of *L. monocytogenes* 4b serotype H7858 following oral infection. a total of 960 mariner mutants were screened by oral inoculation of 6-8 week old Balb/C mice with recovery of output pools at 1-day post-infection. 225 mutants were initially identified as being absent in the output pool of the liver, spleen or mesenteric lymph nodes. These mutants were re-organised into a new pools and 24 mutants were re-infected into the mice per pool. From the second screen 25 mutants were identified as absent in the output pool of the liver, spleen or mesenteric lymph node. The mutation leading to the attenuation was identified by arbitrary PCR, sequencing and comparison with *L* *. moncytogenes* H7858 genome (TIGR or NCBI). To verify that the insertion site was correctly identified a PCR was carried out using primer corresponding to the transposon insertion and primer for gene of interest in all cases (data not shown). Seven mutants were assessed by Southern blot analysis and all demonstrated single transposon insertions in line with previous studies of this system [14,28]. The sequence of the 25 attenuated clones corresponded to 21 distinct loci (Table **2** and Figure **3**). Three of the insertion sites corresponded to a mutation in the internalin A (*inlA*) gene. This is to be expected as *inlA* is important for oral infection and listerial uptake into intestinal epithelial cells [29]. Furthermore this demonstrates the robustness of the STM screen. In line with previous STM mutant studies in *L. monocytogenes* [6] and other pathogens [3,4] we provide a table of the insertion sites for mutants identified in our study (Table **2**) and a brief discussion of the potential role of individual genes in oral infection follows. Physiological analysis of individual mutants was used to provide clues as to stress-related defects which may impact upon gut colonisation. Future work in our laboratory will analyse the impact of precise mutations in these candidate 20 loci upon oral pathogenesis of *L. monocytogenes*.

**Table 2:** Overview of mutants identified from STM mouse screen.

**Gene name**	**Size (kb**)	**Transposon insertion site**	**Initial Screen Liver**	**Initial Screen Spleen**	**Initial Screen Mesenteric Lymph Nodes**	**Annotation**	**Function**
lmOh7858_0215	1.209	855 bp/621bp	Present	Present	Not Detected	ABC transport protein	permease activity
lmOh7858_2367	1.371	572 bp	Present	Present	Not Detected	CBS domain protein	unknown function
lmOh7858_pLM80_0049	0.990	115 bp/150 bp	Present	Present	Not Detected	pil0073	unknown function
lmOh7858_2579	0.972	520 bp	Not Detected	Not Detected	Present	iron ABC transporter	iron ion transport
lmOh7858_0944 (*hemG*)	1.3	1197 bp	Present	Present	Not Detected	protoporphyrinogen oxidase	porphyrin biosynthetic process
lmOh7858_3003	0.69	356 bp	Present	Not Detected	Present	transcriptional regulator	regulatory functions
lmOh7858_2658 (*prfB*)	0.984	390 bp	Present	Present	Not Detected	peptide chain release factor 2	translational termination
lmOh7858_0796	0.405	-100 bp	Present	Present	Not Detected	conserved hypothetical protein	unknown function
lmOh7858_2449 (*gp49*)	0.915	57 bp	Present	Not Detected	Present	protein gp49 homolog	energy metabolism: electron transport
lmOh7858_0586	1.497	941 bp	Present	Present	Not Detected	conserved hypothetical protein	unknown function
lmOh7858_0398	1.395	1011 bp	Present	Present	Not Detected	phosphotransferase system	Fructose specific IIB subunit family
lmOh7858_0671	1770	-0.05bp	Not Detected	Not Detected	Present	LPXTG-motif cell wall anchor domain protein	
lmOh7858_0498 (*inlA*)	1.3	2394 bp	Present	Present	Not Detected	internalin A	Pathogenesis
lmOh7858_2272	1.236	690bp	Not Detected	Present	Present	ABC transport, permease	transport and binding proteins
lmOh7858_0137	0.421	696bp	Not Detected	Not Detected	Present	transcriptional regulator	Regulatory functions:DNA interactions
lmOh7858_1239	1.119	842bp	Present	Not Detected	Present	propanol dehydrogenase	Energy metabolism; propanediol utilization
lmOh7858_1060	1.326	720bp	Not Detected	Present	Present	cation transport protein	cation transmembrane transporter activity
lmOh7858_2098	1.097	250bp	Present	Present	Not Detected	DNA-damage-inducible protein P	
lmOh7858_0898	6.084	300bp	Present	Not Detected	Present	cell wall surface anchor f protein peptidoglycan-based cell wall biogenesis	
lmOh7858_2535	0.474	-0.5bp	Present	Not Detected	Not Detected	Conserved hypothetical protein	Degradation of proteins and peptides

### Genes encoding internalins

In the H7858 4b strain there are a total of 26 genes encoding putative internalins. From the *in vivo* STM screen in mice two internalins genes were identified as having a role in oral infection, *inlA* and lmOh7858_0671. InlA is the best characterized member of the internalin family and mediates recognition and invasion of epithelial cells through specific interaction with host E-cadherin (Ecad) [30]. Therefore the identification of *inlA* from our screen corresponds with earlier findings of the importance of *inlA* for oral infection and verifies that the conditions we used for our screen were appropriate for identification of virulence loci in *L. monocytogenes*.

**Figure 4:**
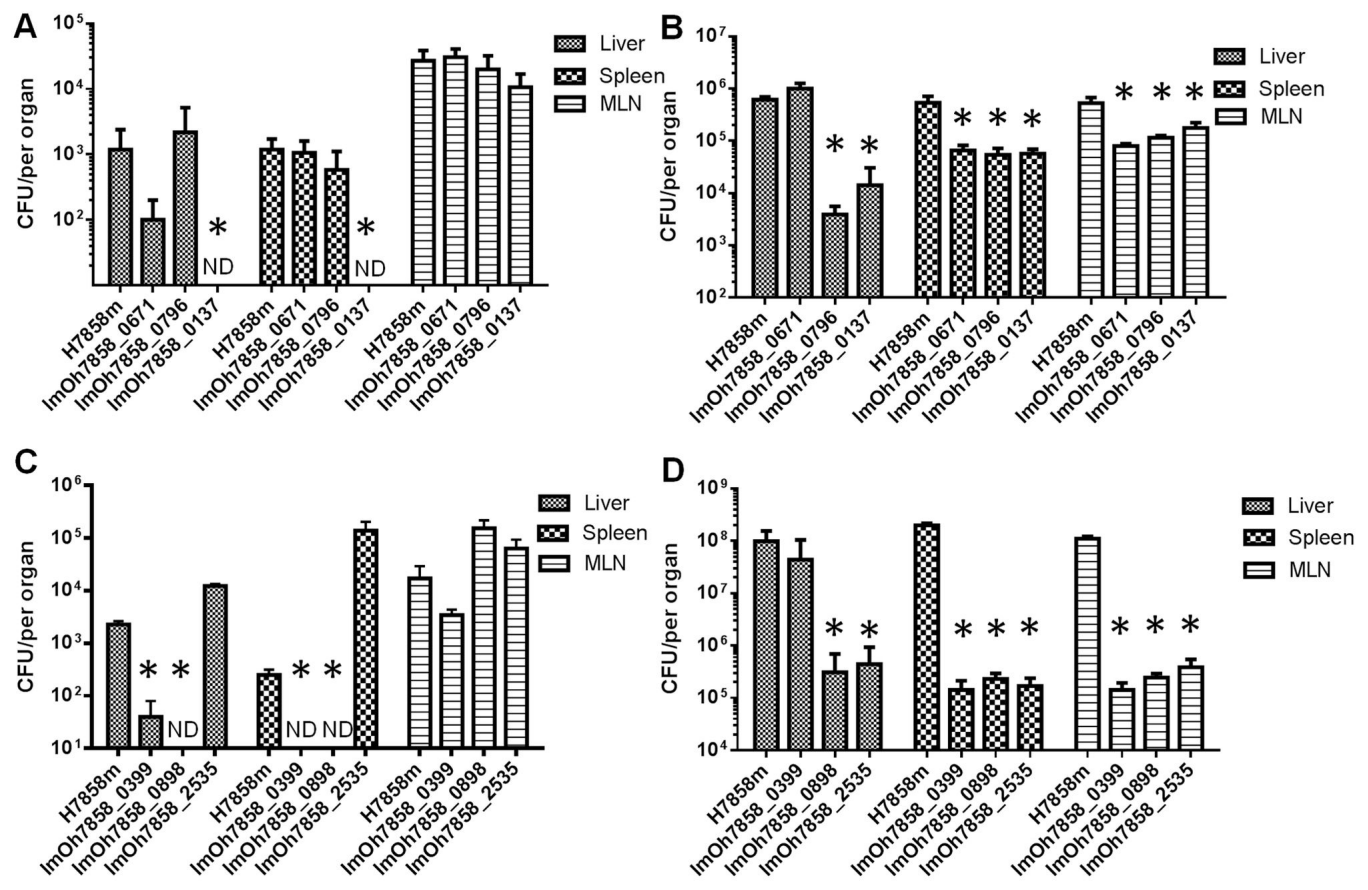
In vivo analyses of individual Tn mutants after oral infection. The kinetics of infection was analyzed on day 1 (A) (C) and day 3 (B) (D) post infection. Bacterial infection was monitored in the liver, spleen and mesenteric lymph nodes. Values are the mean and standard deviation of 5 mice and CFU per organ. ND, not detected. * indicates P<0.05 relative to wild-type control.

The second internalin identified by the screen was lmOh7858_0671 (Figure **3**). This gene is 82% homologous to the EGDe gene lmo0610. This is a LPXTG internalin that contains several other regions such as a signal peptide, 8 leucine rich repeats (LRR), 2 PKD domains and a sorting signal (Figure **S1**). Previous microarray analysis identified that the EGDe homologue lmo0610 is regulated by the alternative sigma factor σ^B^ and contains a σ^B^ promoter sequence upstream from the gene [31]. Clustal W analysis identified that the same σ^B^ promoter sequence was present upstream from the lmOh7858_0671 start site in H7858 indicating that it is most likely regulated by this sigma factor. Significantly, σ^B^ is known to be activated by conditions encountered in the gastrointestinal tract [32]. To verify the results from the initial STM screen this mutant was orally infected into mice and compared to the wild-type H7858^m^. This mutant showed a 1-log decreased infection of the liver at one day post-infection (Figure **4A**) and had 1-log decrease in survival in spleen and MLN on day 3 (Figure **4B**).

### lmOh7858_0898

Another transposon insertion mutant identified in the screen was in lmOh7858_0898 (Figure **3**). This gene encodes a cell wall surface anchor family protein that contains a LPXTG motif, which is the signature sequence that is recognized by the sortase enzyme for localization to the cell wall (Figure **S1**). As well as the LPXTG motif this gene also contains 8 Bacterial-like Ig, which is mostly likely a PKD domain, but it does not contain a LRR region (Figure **S1**). In addition upstream from the start site is a putative PrfA box (TTAAAAATTACTAA) indicating this gene could be regulated by PrfA (Figure **S1**). Interestingly, the homologue of this gene in EGDe (lmo0842) has previously been shown to be upregulated in the host compared to stationary growth in BHI [33]. Furthermore the homologue of this gene was downregulated when grown in soil after 15, 30 minutes and 18 hours (10-fold decreased expression) of exposure to soil [34]. Piveteau and colleagues postulate that virulence associated genes are downregulated due to stimuli in the soil which result in decreased expression of virulence associated genes [34]. When this mutant was subsequently used to orally infect Balb/C mice it had a reduced ability to proliferate in the liver and spleen on day 1 and day 3 post-infection compared to the wild-type strain (Figure **4 C,D**).

### Peptide chain release factor (prfB)

**Figure 5:**
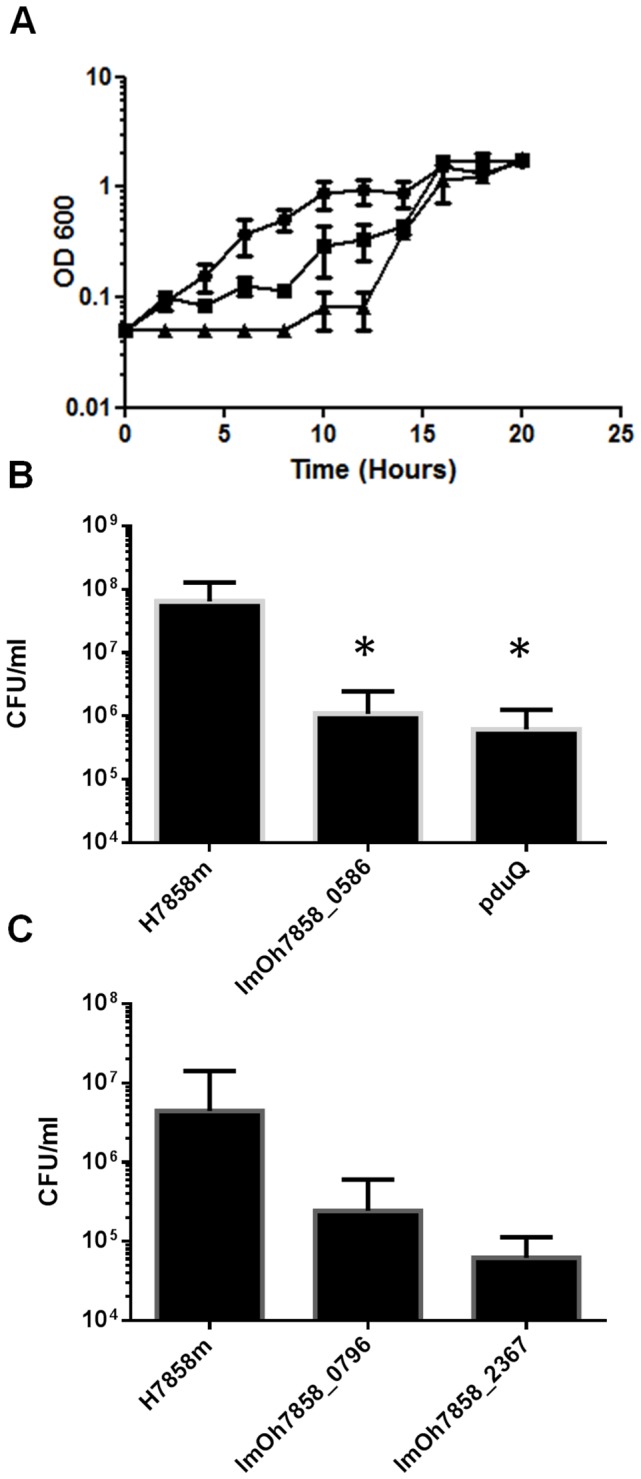
In vitro analysis of virulence-attenuated Tn mutants. All mutants were subjected to in vitro stress analysis as outlined in Materials and Methods. Shown are mutants which differ from the wild-type in an aspect of their stress resistance profile. For clarity mutants with similar profiles to the wild-type are not shown. (A) Growth kinetics of mariner mutants compared to wild-type H7858m grown in BHI + 7.5% NaCl. The mariner mutants in *prfB* and lmoH7858_0137 have a decreased ability to grow in a high salt environment compared to the wild-type strain. (B) Survival of wild-type and mariner mutants exposed to synthetic gastric fluid (pH 2.5) for 2 hours. The mutants in lmOh7858_0586 and *pduQ* had 2-log less survival compared to wild-type strain. (C) Survival of wild-type and mariner mutants in BHI containing 1% bovine bile at pH 5.5. The insertion mutants in lmOh7858_0796 and lmOh7858_2367 exhibited decreased survival compared to the wild-type strain after 6 hours of exposure. All experiments were carried out in triplicate three independent times. The values are the mean and standard deviation. * indicates P<0.05 relative to control.

One of the transposon insertion sites identified in the screen was *prfB* a gene encoding a putative peptide chain release factor (RF2) (Figure **3**). RF2 recognizes the translational stop sites UAA and UGA and is itself regulated through RNA frameshifting events [35]. Recent data suggests that RF2 is important for survival and colonization of the gut by the *E. coli* K12 strain [36,37]. An RF2 mutation in *E. coli* leads to growth inhibition, presumably due to aberrant translational termination events and this may also prevent the strain from being able to colonize the gut [36]. While we did not identify a growth defect in BHI (data not shown) the *prfB* mutant was unable to grow to the same degree as the wild-type in the presence of BHI and high salt (7.5% NaCl) (Figure **5A**). This phenotype may account for the inability of our mutant to survive GI infection, as increased osmolarity of the upper small intestine (equivalent to 0.3 M NaCl) would provide an *in vivo* challenge for this mutant [38].

### lmOh7858_0586

Another interesting locus identified in the STM screen was lmOh7858_0586. This gene is part of a putative operon ranging from lmOh7858_0585 to lmOh7858_0589 (Figure 3). The LmOh7858_0586 gene has 89% homology to the EGDe gene lmo0528, which encodes a hypothetical secreted protein. We show that a transposon insertion in lmOh7858_0586 results in decreased survival in synthetic gastric fluid (SGF) (Figure **5B**). This mutant exhibited a 2-log decrease in survival after 2 hours of exposure to SGF compared to the wild-type H7858^m^ strain [22].

### lmOh7858_2367

Another gene identified from the STM screen was lmOh7858_2367, which encodes a cystathionine-β-synthase (CBS) domain (Figure **3**). Bioinformatic analysis demonstrates that this particular CBS domain is most likely associated with CorC_HlyC transport. This small domain is found in Na^+^/H^+^ antiporters, in proteins involved in magnesium and cobalt efflux, and in association with some proteins of unknown function. When this mutant was exposed to 1% bovine bile at pH 5.5 it resulted in a 1-log decrease in survival compared to the wild-type after 6 hours of exposure (Figure **5C**). There was no effect on the mutant when it was exposed to bile at pH7 (data not shown). This indicates that a transposon insertion at this site affects survival in bile under conditions similar to those encountered in the duodenum where bile mixes with chyme from the stomach [28,39,40].

### ABC transporters

From the STM screen several of the isolates were identified as being ABC transporters. A transposon insertion into the gene lmOh7858_2272 which encodes a putative ABC transporter was identified as affecting gastrointestinal pathogenesis (Figure **3**). This gene is part of an operon with lmOh7858_2271 which is an ABC transporter with an ATP binding protein. From InterPro Scan analysis this ABC transporter is a member of the ABC-2 family of transporters. There are 9 members of this family, which mostly consists of domains of unknown function. LmOh7858_2272 has 93.4% homology to its *L. monocytogenes* EGDe homologue lmo2140.

Transposon insertions into lmOh7858_0215 were independently identified twice in our STM screen. This gene is part of a three-gene operon ranging from lmOh7858_0213 to lmOh7858_0215 (Figure 3). LmOh7858_0215 is an ABC transporter with an FtxS-like permease, which is one of a family of predicated permeases and hypothetical transmembrane proteins that have been shown to transport lipids targeted to the outer membrane (OM) across inner membrane (IM) in Gram negative bacteria.

A transposon insertion in lmOh7858_2579 was identified as reducing oral infection in our mouse model. This gene is part of a three-gene operon (lmOh7858_2579-lmOh7858_2577) that encodes an iron (hemin) ABC transport system (Figure **3**). The gene lmOh7858_2579 has 89.5% homology to the EGDe gene lmo2431 recently demonstrated to be part of the *hupDGC* operon [41,42]. It was established that a mutation in this operon prevents uptake of iron from hemoglobin and hemin and results in significant attenuation in systemic infection in mice [41]. Most iron regulation is under the control of ferric uptake regulator (FUR) and a Fur box is present upstream from *hupD* [43]. An identical FUR box is found upstream from lmOh7858_2579 (Figure **S2**). It was also previously found that the EGDe homologue is upregulated in the host compared to stationary growth in BHI indicating that this gene plays a role *in vivo* [33].

### lmOh7858_0399

lmOh7858_0399 is annotated as a fructose specific IIB subunit (Figure 3) and a component of a putative phosphoenolypyruvate-dependent phosphotransferase (PTS) system [44]. Fructose metabolism has been linked to virulence in other pathogens [45,46,47]. This operon is usually regulated by FruR, which belongs to the DeoR family of transcriptional regulators. Directly upstream from lmOh7858_0400 is a DeoR transcriptional regulator (Figure **3**). More work would have to be carried out to determine how the PTS^Fru^ system may be involved in survival during GI phase of infection. To verify the results from the STM screen this transposon mutant was orally infected into Balb/C mice and shown to have significantly decreased survival on day 1 and day 3 (Figure **4 C,D**). During the early phase of infection there were no detectable mutant bacteria detected in the spleen and a 2-log difference in the amount of bacteria present in the liver compared to the H7858^m^ wild-type. Furthermore this transposon mutant had a decreased ability to proliferate in the spleen and MLN during the late stage of GI infection.

### Protoporphyrinogen oxidase (hemG)

The *hemG* gene (Figure **3**) is a protoporphyrinogen oxidase that is involved in the penultimate step in heme biosynthesis [48,49]. *L. monocytogenes* has all the necessary genes for biosynthesis of heme from glutamate via the C_5_ pathway [50]. In *E. coli* and *Bacillus subtilis* a mutation in *hemG* renders the bacteria heme defective [51,52].

### lmOh7858_1060 (trkH)

On the TIGR website lmOh7858_1060 (Figure **3**) is annotated as a cation transport protein but CDART and InterPro Scan results demonstrate that it has homology to TrkH, a key component in potassium transport in many bacteria [53]. In prokaryotes, K^+^ is essential for the activation of enzymes, for turgor pressure homeostasis, maintaining intracellular pH and for salt tolerance [54,55]. The transposon insertion in lmOh7858_1060 did not affect growth at elevated NaCl concentrations (data not shown). A recent publication identified a *trkH* homologue in the facultative intracellular pathogen *Francisella tularensis* that is involved in systemic dissemination in mice [56].

### lmOh7858_0137

The gene lmOh7858_0137 encodes a protein annotated as a member of the Crp/Fnr family of transcriptional regulators (Figure **3**). Members of the Crp/Fnr superfamily are involved in a vast range of physiological functions such as metabolism, anaerobic and aerobic respiration, resistance to oxidative stress and virulence [57]. A mutant in the lmOh7858_0137 homologue in *L. monocytogenes* strain F2365 (LMOf2365_0130) was previously exposed to several stresses (oxidative stress, regulation of carbohydrate utilization, low temperature, heat resistance) in order to determine its function but it was not affected under any of the conditions tested [57,58]. We carried out similar experiments and found that a transposon insertion in lmOh7858_0137 led to a growth defect in a high salt environment (Figure **5A**). *In vivo* analyses in mice indicated that this mutant was not detectable in liver and spleen on day 1 post-infection (Figure **4A**) and on day 3 it had a 3-log difference in survival in liver and 1-log difference in spleen and MLN compared to wild-type (Figure **4B**).

### pduQ

The gene lmOh7858_1239 encodes *pduQ* and a transposon insertion into this gene was identified in our STM screen as impacting upon virulence (Figure 3). *PduQ* is involved in degradation of 1,2-propanediol (1,2-PD). It is a propanol dehydrogenase that converts propionaldehyde to propanol [59]. The genes for degradation of 1,2-PD are conserved in three significant food-borne pathogens (*L. monocytogenes, Clostridium perfringens* and *Salmonella* *typhimurium* ) but are absent in almost all other species [60]. Korbel and colleagues have postulated that 1,2-PD is a crucial genomic determinant of pathogenicity associated with food poisoning, by promoting anaerobic growth both in the host and in processed food [60]. In *Salmonella* 1,2-PD was shown to play a role in pathogenesis and a deletion of the *pdu* genes specifically impairs growth in the host [61].

Our data demonstrate that a transposon insertion in *pduQ* results in a 2-log decrease in survival in SGF compared to the wild-type strain indicating the 1,2-PD may be important for survival within the stomach (Figure 5b). Recent work in Salmonella has demonstrated that a *pduA* mutant has low colonization of the chicken cecum which is weakly acidic (pH 6.5) [62]. In addition their work demonstrated increased expression of *pdu* genes in the chicken intestine after infection with Salmonella indicating the importance of these genes in Salmonella virulence [62].

### lmOh7858_2098

lmOh7858 _2098 (Figure 3) is annotated as a DNA-damage-inducible protein P and is homologous to the *dinB* gene originally identified in *E. coli*. However *dinB* mutation in other bacteria such as *E. coli* and *Mycobacterium* failed to exhibit a clear phenotype with respect to survival following exposure to DNA-damaging stressors [63,64]. Similarly when we exposed the transposon mutant to these stresses *in vitro* it did not demonstrate any alteration in survival compared to wild-type strain (data not shown). Further work is needed to fully determine the effect of mutation upon survival *in vivo*.

### Miscellaneous genes

From our STM screen the location of two transposon insertions corresponded to lmOh7858_pLM80_0049 (Figure **3**). This gene is present on the plasmid pLM80 found in *L. monocytogenes* H7858. This plasmid is approximately 80 kb in size and contains several different transposable elements that are not present on the chromosome suggesting that the plasmid is a recent acquisition [65]. The plasmid has a high level of sequence and gene organization homology to the *L. innocua* CLIP 11262 plasmid pLI100 and the *B. anthracis* plasmid pXO2 [66]. The gene in question has a homologue on the pLI100 plasmid from *L. innocua* (pil0073). Both genes are classified as conserved hypothetical genes with no known function. This gene is also part of a 3-gene operon and these genes are also annotated as conserved hypothetical genes (Figure **3**). The mutant was exposed to several environmental stresses (low pH, bile and high salt) and did not demonstrate any discernible phenotype (data not shown). Therefore it is difficult to determine how this gene may play a role in the GI phase of infection.

The gene lmOh7858_2449 was identified in the STM screen (Figure **3**). This gene has homology to gp49 from the *Listeria* bacteriophage A118. The function of the Gp49 protein is predicted to involve endonuclease VII activity, which is the first step in the mismatch repair pathway in T4 bacteriophage [67]. This gene has 62.5% homology to the DNaD gene in the *L. monocytogenes* strain F6854 and the gene is required for replication initiation. When this mutant was exposed to environmental stress (low pH, bile at low pH, high salt) it did not demonstrate any decrease in survival or growth (data not shown).

Transposon insertion into lmOh7858_0796 was identified by the STM screen as affecting virulence. This gene is a hypothetical gene with homologues in other *L. monocytogenes* strains as well as *L* *. welshimeri* and *L. innocua*. Our mutant had decreased survival in BHI containing 1% bovine bile (pH 5.5) (Figure **5C**). Compared to the wild-type the lmOh7858_0796 transposon mutant had a 2-log reduced level of survival after 6 hours of exposure to bile. *In vivo* analyses of this mutant demonstrated that it had decreased survival in liver, spleen and MLN 3-days post-infection compared to H7858^m^ (Figure **4B**). The greatest decrease was seen in the liver with a 3-log decrease in infection.

lmOh7858_3003 (Figure **3**) is classified as belonging to the Sir2 family of transcriptional regulators. Silent information regulator-like proteins (Sir/sirutins) were first identified in *Saccharomyces cerevisiae* and shown to function as transcriptional repressors of telomeres, the silent mating-type loci and ribosomal DNA [68].

From the STM screen two independently isolated mutants of interest corresponded to transposon insertions into lmOh7858_2535. This gene is not on an operon and is classified as having homology to *B. subtilis* YuiD protein (Figure **3**). From bioinformatic analysis it was determined that this gene is related to the acid phosphatase/vanadium-dependent haloperoxidase whose function is currently uncharacterized but it is thought may play a role in phospholipid metabolism [69]. This gene shares 99.4% homology to the EGDe gene lmo2485. From a previous microarray analysis this gene was shown to upregulated more than 2-fold in the host compared to stationary and exponential growth in BHI [33]. Furthermore the gene was classified as being involved in the stress response [33]. When we infected mice with this mutant via the oral route it demonstrated a decreased ability to survive and proliferate in the liver, spleen and MLN during the late stage of GI infection (Figure **4D**).

## Conclusions

We have engineered an improved STM system for the analysis of genetic loci required for intragastric infection by *L. monocytogenes* in the mouse model. The basis of the approach is a mariner transposon system and the method employed a murinized strain of serotype 4b *L. monocytogenes* that is optimized for oral infection in mice. Very recent sequence-based approaches for functional genetic analysis of mutant banks (such as TraDIS) offer great potential for large-scale mutant screening [7]. However these approaches also currently have limitations such as the requirement for complete unbiased transposon coverage, the need for an animal model capable of extremely efficient gastrointestinal colonization/infection, high costs associated with sequencing input and output banks and the inability to work with individual mutants isolated using the system [7]. In contrast STM offers the ability to tailor the size of the input pool to overcome any limitations associated with the animal model and to analyse individual mutants in vitro subsequent to the screen [4,7]. Here we demonstrate that our novel system has identified transposon insertion mutants that are compromised for infection via the oral route. In an approach used previously in *V. cholerae* we also performed analysis of our mutants for resistance to physico-chemical stressors encountered *in vivo* [4]. Some of the mutants identified using our screen were also analyzed for individual infection dynamics in subsequent infection studies. The approach identified an insertion into known virulence-related loci (*inlA, hupDGC*) as well as transposon insertions into genes which encode another internalin, a transcriptional regulator and genes putatively involved in metabolic processes (including (putatively) fructose metabolism and propanol metabolism). Analysis of the roles of these loci in pathogenesis will form the basis of further study.

## Supporting Information

Figure S1 **Characterisation of internalins from STM screen.** (a) Genomic organization of inlA and insertion site in transposon mutants identified in STM screen in mouse model of infection. The diagram was drawn approximately to scale using *Listeria monocytogenes* H7858 genome sequence data (TIGR). Open reading frames (shaded in gray) are genes with transposon insertion. Black arrowheads represent the approximate location of transposon insertion. White open reading frames are flanking genes. Lollipops indicate predicted terminator locations. (b) Schematic domain organisation of internalin lmOh7858_0671 based on EGDe homologue lmo0610 and InterPro Scan. Black box represent the signal peptide, pink box the 8 LRR, green region 2 PKD domains, yellow arrow sorting signal and yellow box the LPXTG motif. Upstream from start site is the σ^B^ promoter region at 61 bp and 82 bp from start site. (c) Schematic domain organization of lmOh7858_0898 based on Interpro Scan results. Black box represents a domain of hypothetical protein PA1324 superfamily, green box 8 PKD and yellow box represents LPXTG domain. Approximatley 199 bp upstream from start site there is a putative PrfA box.
(PPTX)

Figure S2 **Clustal W analysis of FUR box found upstream of lmOh7858_2579.** This region was compared to FUR box found in *hupD* homologue in EGDe and found to be completely identical to FUR box found in *hupD* region.
(PPTX)

Table S1 **Primers used in this study.**
(DOCX)
