# Supplementary material for: A Mariner Transposon-Based Signature-Tagged Mutagenesis System for the Analysis of Oral Infection by Listeria monocytogenes
Source: PLoS One. 2013 Sep 12;8(9):e75437. doi: 10.1371/journal.pone.0075437 (PMC3771922; doi:10.1371/journal.pone.0075437)
Supplement: Figure S1 — Characterisation of internalins from STM screen. (a) Genomic organization of inlA and insertion site in transposon mutants identified in STM screen in mouse model of infection. The diagram was drawn approximately to scale using Listeria monocytogenes H7858 genome sequence data (TIGR). Open reading frames (shaded in gray) are genes with transposon insertion. Black arrowheads represent the approximate location of transposon insertion. White open reading frames are flanking genes. Lollipops indicate predicted terminator locations. (b) Schematic domain organisation of internalin lmOh7858_0671 based on EGDe homologue lmo0610 and InterPro Scan. Black box represent the signal peptide, pink box the 8 LRR, green region 2 PKD domains, yellow arrow sorting signal and yellow box the LPXTG motif. Upstream from start site is the σB promoter region at 61 bp and 82 bp from start site. (c) Schematic domain organization of lmOh7858_0898 based on Interpro Scan results. Black box represents a domain of hypothetical protein PA1324 superfamily, green box 8 PKD and yellow box represents LPXTG domain. Approximatley 199 bp upstream from start site there is a putative PrfA box. (PPTX) [file pone.0075437.s001.pptx]

## Slide 1
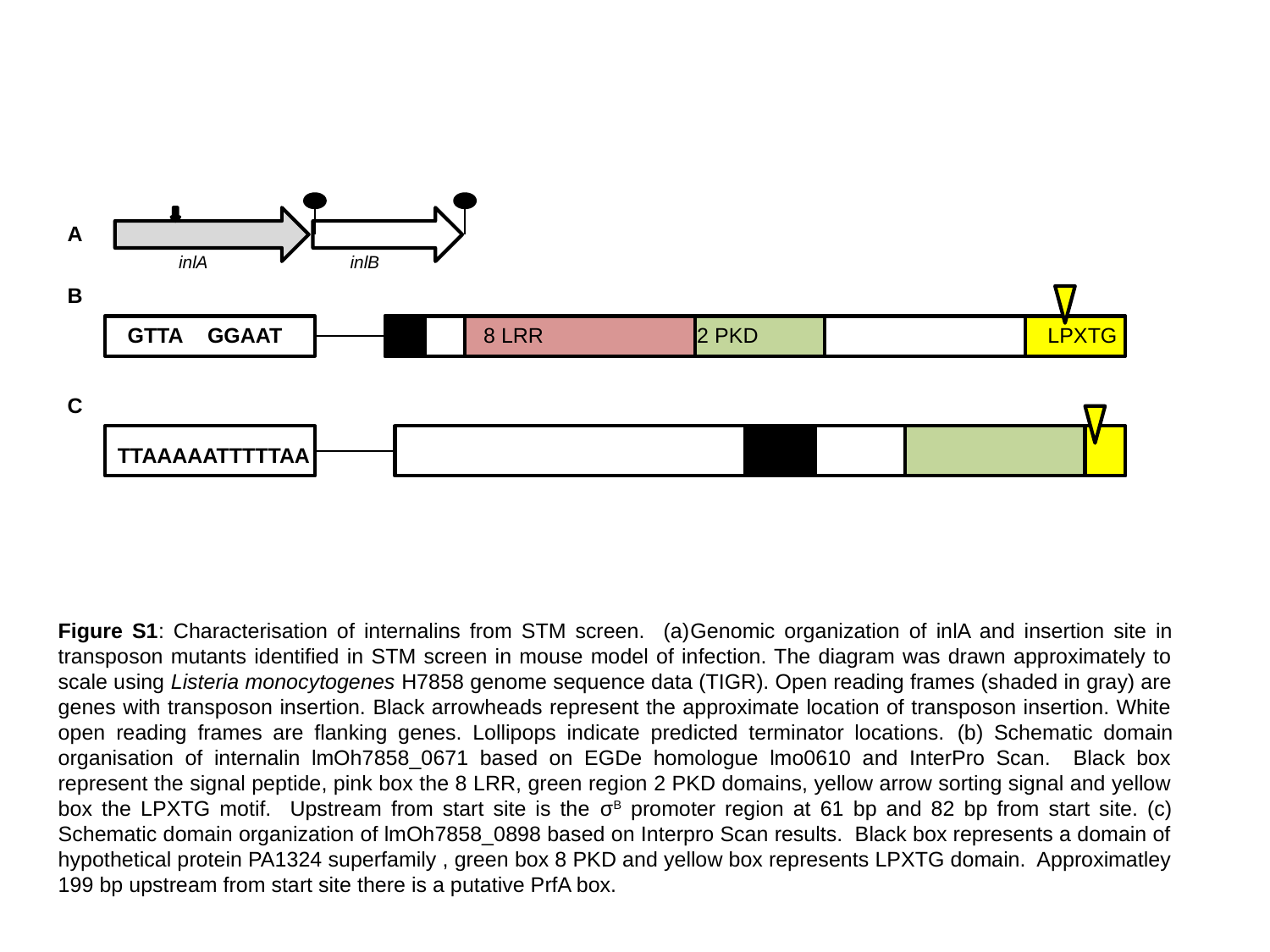

inlA
 inlB
A
B
GTTA
GGAAT
 8 LRR 2 PKD LPXTG
C
TTAAAAATTTTTAA
Figure S1: Characterisation of internalins from STM screen. (a)Genomic organization of inlA and insertion site in transposon mutants identified in STM screen in mouse model of infection. The diagram was drawn approximately to scale using Listeria monocytogenes H7858 genome sequence data (TIGR). Open reading frames (shaded in gray) are genes with transposon insertion. Black arrowheads represent the approximate location of transposon insertion. White open reading frames are flanking genes. Lollipops indicate predicted terminator locations. (b) Schematic domain organisation of internalin lmOh7858_0671 based on EGDe homologue lmo0610 and InterPro Scan. Black box represent the signal peptide, pink box the 8 LRR, green region 2 PKD domains, yellow arrow sorting signal and yellow box the LPXTG motif. Upstream from start site is the σB promoter region at 61 bp and 82 bp from start site. (c) Schematic domain organization of lmOh7858_0898 based on Interpro Scan results. Black box represents a domain of hypothetical protein PA1324 superfamily , green box 8 PKD and yellow box represents LPXTG domain. Approximatley 199 bp upstream from start site there is a putative PrfA box.
