# Supplementary material for: A Mariner Transposon-Based Signature-Tagged Mutagenesis System for the Analysis of Oral Infection by Listeria monocytogenes
Source: PLoS One. 2013 Sep 12;8(9):e75437. doi: 10.1371/journal.pone.0075437 (PMC3771922; doi:10.1371/journal.pone.0075437)
Supplement: Figure S2 — Clustal W analysis of FUR box found upstream of lmOh7858_2579. This region was compared to FUR box found in hupD homologue in EGDe and found to be completely identical to FUR box found in hupD region. (PPTX) [file pone.0075437.s002.pptx]

## Slide 1
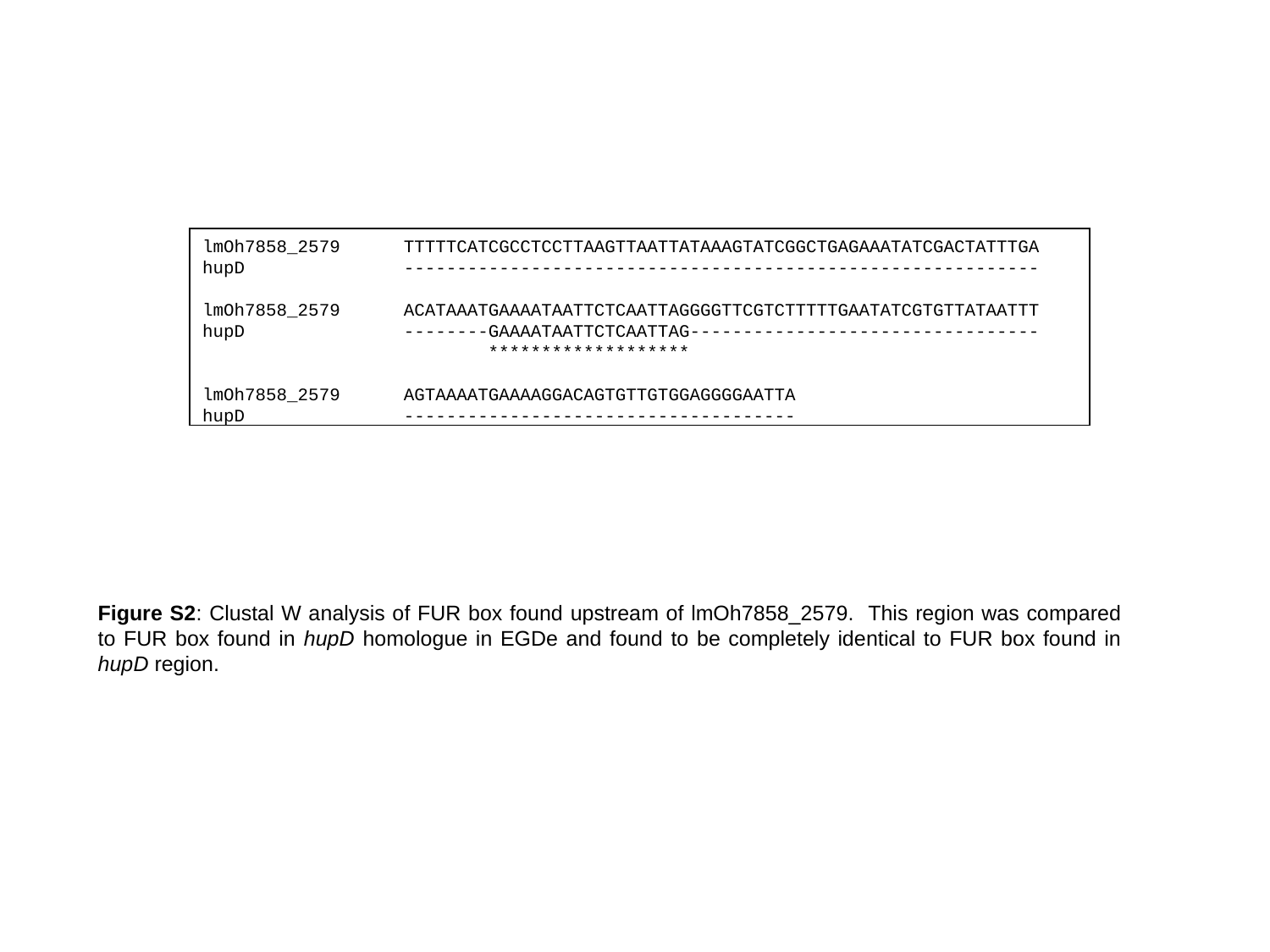

lmOh7858_2579 TTTTTCATCGCCTCCTTAAGTTAATTATAAAGTATCGGCTGAGAAATATCGACTATTTGA
hupD ------------------------------------------------------------
lmOh7858_2579 ACATAAATGAAAATAATTCTCAATTAGGGGTTCGTCTTTTTGAATATCGTGTTATAATTT
hupD --------GAAAATAATTCTCAATTAG---------------------------------
	 *******************
lmOh7858_2579 AGTAAAATGAAAAGGACAGTGTTGTGGAGGGGAATTA
hupD -------------------------------------
Figure S2: Clustal W analysis of FUR box found upstream of lmOh7858_2579. This region was compared to FUR box found in hupD homologue in EGDe and found to be completely identical to FUR box found in hupD region.
