# Supplementary material for: A Mariner Transposon-Based Signature-Tagged Mutagenesis System for the Analysis of Oral Infection by Listeria monocytogenes
Source: PLoS One. 2013 Sep 12;8(9):e75437. doi: 10.1371/journal.pone.0075437 (PMC3771922; doi:10.1371/journal.pone.0075437)
Supplement: Table S1 — Primers used in this study. (DOCX) [file pone.0075437.s003.docx]

Table S1: Primers used in this study

| **Primers** | **Sequence (5’-3’)^a^** | **Reference** |
| --- | --- | --- |
| IM490 (Chromosome-F) | ATAT**CCATGG**AAAAGGAGTGTATATA***GTG***AGAAAAAAACGATATGTATGG *Nco*I | [[1](#_ENREF_1)] |
| IM466 (Chromosome-R) | ATAT**CTGCAG**CAAACGTTGCTGTATAGCTATTGG *Pst*I | [[1](#_ENREF_1)] |
| IM317 (inlA muta-F) | AAAC**AGATCT**AGACCAAGTTACAACG *Bgl*II | [[1](#_ENREF_1)] |
| IM318 (inlA muta-R) | AATT**CCA**CTTCTT**TGG**TTGTTTCTTTGC *Bst*XI | [[1](#_ENREF_1)] |
| RT1 | ATCGTCGA**CTCGAG**TACAACCTC**GGCGCC**[NK]_20_**GGCGCC**GGTTAGAATG**CTCGAG**CATTAGTC*Xho*I, *Nar*I | This study |
| J3 | ATCGTCGA**CTCGAG**TACAACCTCGGCGCC *Xho*I | This study |
| J4 | GACTAATG**CTCGAG**CATTCTAACC *Xho*I | This study |
| pJZ037 FP | TGACAGCTTCCAAGGAGCTAA | This study |
| pJZ037 RP | CCCCGGTCTCTAGACCCTAT | This study |
| Marq207 | GGC CAC GCG TCG ACT ACT CAN NNN NNN CTA AT | [[2](#_ENREF_2)] |
| Marq 208 | GGC CAC GCG TCG ACT ACG AC | [[2](#_ENREF_2)] |
| JZ-001 | CTA TGA GTC GCT TTT GTA AAT TTG GAA AGT TAC ACG TTA CTA A | This study |
| JZ-002 | TAA AGA GGT CCC TAG CGC CTA CGG GGA ATT TGT ATC GAT | This study |
| JZ-003 | GAG TGG GGT ACG CG AAT ACG | This study |
| JZ-184 | GACTTATCGGCCAACCT | (6) |
| JZ-185 | TACGCACCGGACGAG | (6) |

Underlining and bold indicates restriction enzyme sites. NK represents nucleotides N; A,C,T,G and K; G or T.

1. Monk IR, Gahan CG, Hill C (2008) Tools for functional postgenomic analysis of listeria monocytogenes. Appl Environ Microbiol 74: 3921-3934.

2. Monk IR, Casey PG, Hill C, Gahan CG Directed evolution and targeted mutagenesis to murinize Listeria monocytogenes internalin A for enhanced infectivity in the murine oral infection model. BMC Microbiol 10: 318.

3. Leenhouts K, Buist G, Bolhuis A, ten Berge A, Kiel J, et al. (1996) A general system for generating unlabelled gene replacements in bacterial chromosomes. Mol Gen Genet 253: 217-224.

4. Maguin E, Duwat P, Hege T, Ehrlich D, Gruss A (1992) New thermosensitive plasmid for gram-positive bacteria. J Bacteriol 174: 5633-5638.

5. Zemansky J, Kline BC, Woodward JJ, Leber JH, Marquis H, et al. (2009) Development of a mariner-based transposon and identification of Listeria monocytogenes determinants, including the peptidyl-prolyl isomerase PrsA2, that contribute to its hemolytic phenotype. J Bacteriol 191: 3950-3964.

6. Cao M, Bitar AP, Marquis H (2007) A mariner-based transposition system for Listeria monocytogenes. Appl Environ Microbiol 73: 2758-2761.
